# Pseudoangiomatous stromal hyperplasia (PASH) of the breast: A case series

**DOI:** 10.1007/s13304-024-02013-z

**Published:** 2024-10-04

**Authors:** Eduardo Spina, Emanuela Esposito, Claudio Siani, Valeria Varone, Gerardo Ferrara, Raimondo di Giacomo

**Affiliations:** 1 Department of Clinical Medicine and Surgery, University of Naples Federico II, Naples, Italy; 2 Breast Surgery Division, Istituto Nazionale Tumori-IRCCS-Fondazione G. Pascale, Naples, Italy; 3 Pathology Division, Istituto Nazionale Tumori-IRCCS-Fondazione G. Pascale, Naples, Italy

**Keywords:** Breast, Surgery, Pseudoangiomatous stromal hyperplasia, Pathology, Surgical excision

## Abstract

Pseudoangiomatous stromal hyperplasia (PASH) is a benign mesenchymal proliferative lesion of the breast. In 2005, only 109 cases have been reported since its initial description in 1986 by Vuitch et al. when it presented in one patient as a palpable breast mass. We retrospectively reviewed data from 2020 to 2023 of patients diagnosed with PASH by surgical excision. Our 13 cases represent one of the most numerous reported from a single institution. All histologic specimens were examined by a single pathologist. All patients had breast masses on imaging or were clinically evident. Eleven of the patients (84.6%) were diagnosed by surgical excision, whilst only two (15.4%) were diagnosed by core needle biopsy. Imaging revealed no strongly distinctive features for PASH. The age of the patients ranged from 25 to 68 years. All but one of the women were premenopausal at the time of diagnosis. This study suggests that PASH is a lesion whose diagnosis is often incidental and the recommended treatment is more commonly surgical.

## Introduction

Pseudoangiomatous stromal hyperplasia (PASH) is a rare and benign proliferative mesenchymal breast lesion. Its clinical and radiological outcomes, as well as the optimal treatment, remain unclear. It was first described in 1986 by Vuitch et al. [1] in nine patients with nodular mass lesions and a histological pattern of vasoproliferative proliferation. By 2008, only 109 cases had been reported in international journals since 1986 [2, 3]. PASH is often seen as a microscopic lesion detected incidentally [2], rarely as a clinically palpable mass with a different diagnosis, or as a giant mass causing breast asymmetry [2, 4, 5]. The clinical and radiological characteristics of PASH often lead to misdiagnosis as fibroadenoma, phyllodes tumor or hamartoma [6–8]. Initially considered a variant of mammary hamartoma, PASH is now recognized as a benign proliferation of stromal myofibroblasts. There is no established conservative treatment for PASH. According to Kwang Hyun Yoon et al. [23], the choice of treatment for PASH is determined by the medical doctor’s preference, based on core needle biopsy (CNB) results, imaging, and clinical presentation. Treatment options are divided into observation (routine surveillance), vacuum-assisted excision (VAE), or surgical excision [23]. It has also been reported in the literature that stromal cells in PASH are positive for the progesterone receptor [16]. These findings support the hypothesis that PASH is a hormone-dependent proliferation of intralobular stromal cells and may respond to tamoxifen, although the effect might be observed only with prolonged therapy. Long-term tamoxifen may not be ideal due to its side effects [10, 22] and may be inappropriate in young premenopausal women [10, 13, 22]. Yoon et al. described a disease progression in 11 cases (16.6%), with a median progression period of 26 months (ranging from 6 to 36 months) after the initial treatment [23]. Furthermore, there is a reported case of malignant transformation of PASH in the literature, and some cases of PASH are reported in association with concurrent neoplasms [13, 14, 20]. The histological appearance is characterized by angular, slit-like interanastomotic spaces lined by thin spindle cells and surrounded by dense collagen stroma. These myofibroblast-lined clefts may be a fixation artifact caused by retraction of collagen stroma [9, 11, 12]. Although there are no red blood cells in these spaces, PASH is occasionally misdiagnosed as low-grade angiosarcoma [2, 16, 20]. The purpose of this paper is to review the clinical and radiological characteristics of PASH, adding our Institute’s experience, with the aim of helping to define a standardized approach for the diagnosis and management of PASH.

## Materials and methods

This study has been reported in line with the PROCESS criteria [24].

In this study, we conducted a search of the pathology database for all cases diagnosed as PASH on surgical excisions from January 2020 to July 2023. Patient medical records were retrospectively reviewed for information regarding patient demographics, contraceptive and/or hormone replacement therapy history, personal and family history of cancer, lesion presentation, clinical diagnosis, imaging and histopathological diagnosis. Clinical and imaging follow-up data were also recorded.

Mammographic and ultrasound imaging were reviewed for all patients. All samples hematoxylin and eosin-stained were examined by a single pathologist with long-standing expertise in breast pathology. The diagnosis of PASH was mostly associated with stromal fibrosis, sclerosing adenosis, fibroadenomatous hyperplasia and atypical ductal hyperplasia. Patients over 40 years of age were evaluated with both mammography and ultrasound, while those who were younger than 40 years were assessed with ultrasound alone. All patients underwent ultrasound-guided core biopsy. The diagnoses of PASH by core needle biopsy were two (15.4%). All patients were treated with wide local excision. All the patients signed an informed consent for the surgical procedure. Follow-up procedures, initially at 6 months after surgery and then annually, included clinical examination, breast ultrasound and mammography for patients aged 40 and above. For patients under 40 years of age, follow-up included only clinical breast examination and ultrasound at 6 months after surgery, followed by annual checks.

## Results

**Fig. 1:**
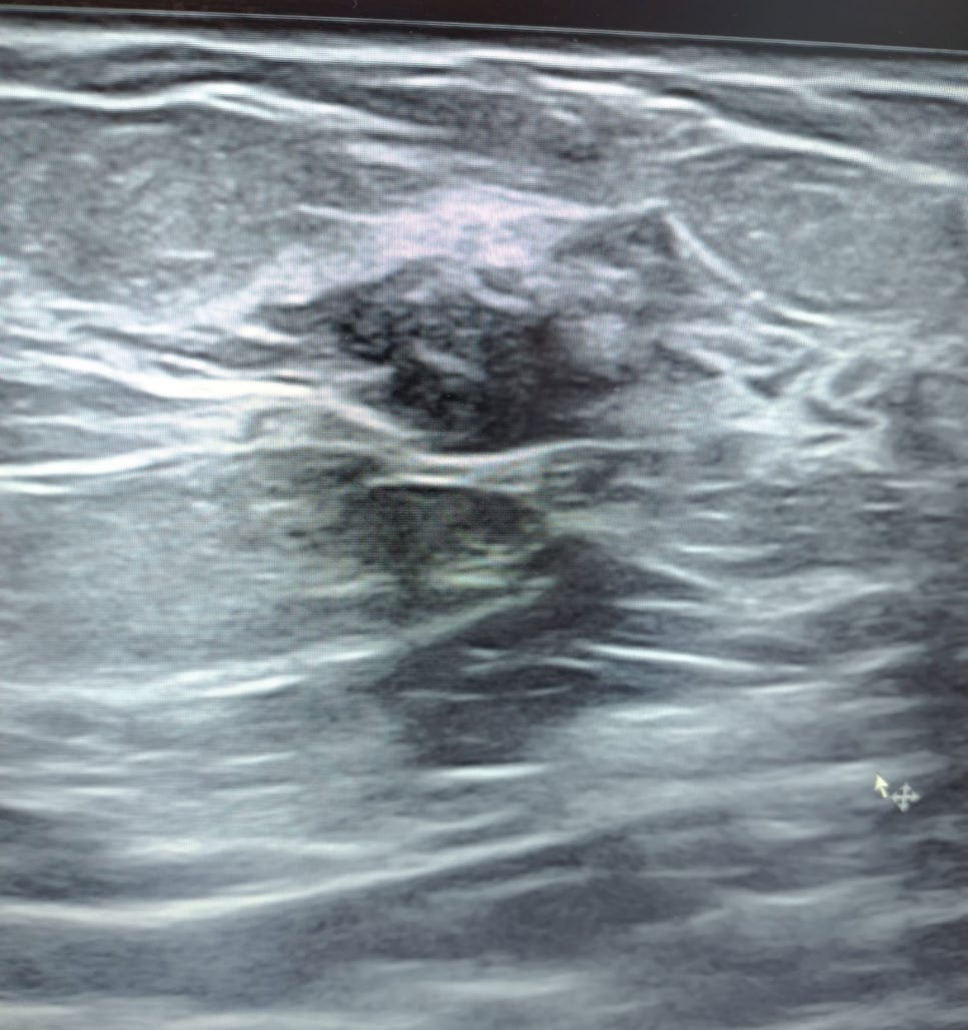
Ultrasound scan of PASH

Between January 2020 and July 2023 at the National Cancer Institute of Naples "G. Pascale", approximately 800 radiologically guided breast biopsies were performed. 2 lesions out of 800 were characterized as cases of PASH after core needle biopsy, whilst 11 out of 13 were diagnosed after surgery. All patients diagnosed with PASH were female; 3 out of 13 patients (23,1%) were postmenopausal. None of the postmenopausal patients had received hormone replacement therapy. Only four patients (30,8%) had a family history of breast cancer. The average age of patients with PASH was 42.5 years (range: 25–68 years). A palpable and painless breast mass was found in ten patients (76,9%) during clinical examination. Three patients presented with non-palpable lesions (15,4%) diagnosed by combination of mammography and ultrasound scan. Ultrasound scan described round or oval, heterogeneous, hypoechoic lump with regular margins and no posterior acoustic enhancement in six patients (46,1%) (Fig. 1).

In one third of cases (30,8%) round or oval, heterogeneous, hypoechoic masses with clusters of microcalcifications were described. Only two patients (15,4%) presented with a heterogeneous hypoechoic lesion with irregular margins, and these two patients had pre-operative diagnoses of PASH. According to the American College of Radiology Breast Imaging Reporting and Data System (BI-RADS), two lesions were classified as BIRADS-4 (15,4%) due to mixed echotexture and irregular margins. The remaining 11 lesions (76,9%) were classified as BIRADS-3.

Mammographic examination was performed for ten patients, and only in two of them the diagnosis of PASH was not incidental. The mammographic findings in these two patients with non-incidental PASH were a solid and well-circumscribed radiopaque lesion. In the mammographic findings of incidentally detected PASH, four (30,8%) patients had an irregularly shaped mass with spiculated margins and pleomorphic microcalcifications. The remaining patients (30,8%) had nonspecific mammographic findings (Table 1). Magnetic resonance imaging was performed amongst four patients with dense breasts and limited diagnostic definability.

**Table 1:** Main characteristics

	Age	Dimension (mm)	Palpability	Breast pain	Clip	Mammography	US	Pre-operative diagnosis
N. 1	44	80 mm	+	+		Nonspecific mammographic findings	Regular margins	
N. 2	49	100 mm	+	+		Irregularly shaped mass with spiculated margins and pleomorphic microcalcifications	Regular margins	
N. 3	46	50 mm	+			Solid radiopaque lesion	Irregular margins	+
N. 4	42	12 mm			+	Solid radiopaque lesion	Irregular margins	+
N. 5	25	60 mm	+				Regular margins	
N. 6	31	15 mm			+		Regular margins	
N. 7	68	4 mm			+	Irregularly shaped mass with spiculated margins and pleomorphic microcalcifications		
N. 8	43	40 mm	+			Irregularly shaped mass with spiculated margins and pleomorphic microcalcifications		
N. 9	45	40 mm	+			Irregularly shaped mass with spiculated margins and pleomorphic microcalcifications		
N. 10	49	30 mm	+	+		Nonspecific mammographic findings		
N. 11	33	30 mm	+				Regular margins	
N. 12	46	30 mm	+			Nonspecific mammographic findings	Regular margins	
N. 13	64	30 mm	+			Nonspecific mammographic findings	Regular margins	

Three out of 13 non-palpable lesions were marked by magnetic seed and removed with magnetic-assisted surgery through periareolar incision. Ten out of 13 lesions were removed with wide local excision. All the above-mentioned lesions were completely excised with clear margins. The final histological examination reported 12 (92,3%) multifocal areas of PASH within the same mass and only one was unifocal (7,7%) sized 4 mm. On histological examination, the presence of PASH was associated to fibroadenomata in 2 patients and other benign proliferative changes without atypia amongst 11 patients.

The follow-up is ongoing and none of the patients experienced PASH recurrence to date.

## Pathology

The histological appearance of PASH is characterized by interanastomotic spaces that are angular and slit-like, lined with thin spindle cells and surrounded by dense collagen stroma. These myofibroblast-lined clefts may be a fixation artifact caused by collagen stroma retraction [9, 11, 12].

On pathological examination, the breast masses are generally unilateral, well-circumscribed, and have well-defined and smooth outer surfaces. The cut surface is solid, gray-white. No bleeding or necrosis is observed.

**Fig. 2:**
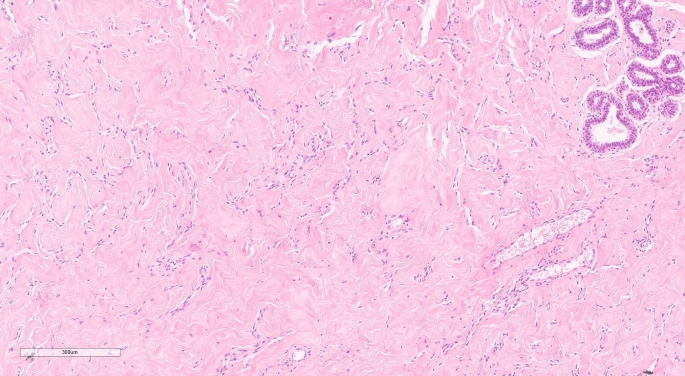
Numerous anastomosing slit pseudoangiomatous spaces lined by spindle cells and separated by thick hyalinized collagen bundles. H and E, 10x

The histopathological examination of the lesion shows complex interanastomotic spaces in a dense collagen stroma, resembling keloids (Fig. 2).

**Fig. 3:**
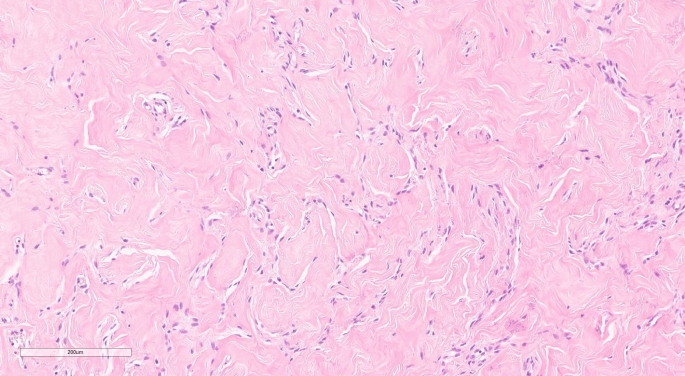
Inter-anastomosing slit-like spaces separating keloid-like collagen. Slit-like spaces have spindle cells (myofibroblasts) at the margins that simulate endothelial cells. H and E, 20x

Some of these spaces have spindle-shaped myofibroblasts at their margins, simulating endothelial cells, but these spaces are usually empty, with no red blood cells, so they are not true vascular spaces (Fig. 3).

**Fig. 4:**
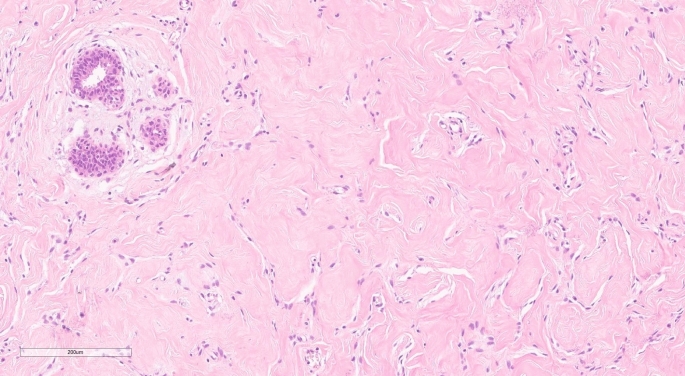
Columnar cell lesion showing typical apical snouts in columnar cells and PASH-like stroma. H and E, 20x

Often, cellular areas or spindle cells can obscure the pseudoangiomatous structure. Epithelial changes (similar to gynecomastia; columnar cell lesions) can be associated, and multinucleated giant cells are rarely reported (Fig. 4).

No mitotic figures, necrosis, or atypia are observed [2, 16, 20]. In some cases, the lesions were more cellular: fascicular PASH is a cellular variant in which myofibroblasts aggregate into bundles with reduced or absent clefts, similar to myofibroblastoma [17–19, 21].

The differential diagnosis includes low-grade angiosarcoma, myofibroblastoma, and fibromatosis. For the differential diagnosis, immunohistochemical studies are performed. PASH lesions were positive for CD34 and Smooth Muscle Actine (SMA), while they were negative for CD31 and desmin. Furthermore, other lesions associated with PASH were benign non-proliferative changes without atypia in all patients.

## Discussion

Pseudoangiomatous stromal hyperplasia (PASH) is a rare and benign proliferative mesenchymal lesion of the breast. Its clinical and radiological outcomes, as well as the optimal treatment have not been clarified. By 2008, only 109 cases had been reported in international journals since 1986 [2, 3]. The clinical and radiological characteristics of PASH often lead to misdiagnosis in the pre-diagnostic phase, considering this lesion as fibroadenoma, phyllodes tumor, or hamartoma [6–8]. Initially considered a variant of mammary hamartoma, PASH is now accepted as a benign proliferation of stromal myofibroblasts but there is no established conservative treatment for PASH.

Based on the studies available in the literature, if the diagnosis of PASH is made through core needle biopsy (CNB), surgical excision may not be necessary, and careful observation with serial mammograms to assess growth over a specified time interval may be considered appropriate [4, 8, 12, 15]. This study suggests that PASH is mainly diagnosed amongst premenopausal and perimenopausal women. We accounted only one case of PASH diagnosed in postmenopausal. There is strong clinical evidence that there is a hormonal basis for the development of PASH.

In the current literature, the treatment for PASH is determined by the physician’s preference based on CNB results, imaging, and clinical presentation. Treatment options are divided into surveillance, vacuum-assisted excision (VAE), or surgical excision [28]. In cases of long-term observation, disease progression 2 years after initial diagnoses has been observed in about 10 to 20% of patients [23–25]. In a multivariate logistic regression analysis, adjusted for CNB results, symptoms, and lesion size, these factors were considered independent factors for progression [23]. Although mass lesions in PASH often grow over time and may recur, they are neither associated with malignancy nor considered to be premalignant lesions [25].

## Conclusions

PASH is a benign breast lesion and it can progress. Based on the results of core biopsies, we suggest surgical excision over observation, especially when symptomatic. We justify this therapeutic choice because of the high growth rate of lesions, which can lead to painful symptoms, become clinically evident, and cause aesthetic discomfort to patients. Another reason supporting surgical removal is the common occurrence of multifocality in PASH lesions, a characteristic that frequently complicates preoperative diagnosis. As showed in our case series, the lesion appeared as a 4 mm mass in only one instance, while in the majority of cases, it manifested as multiple foci of PASH within a larger mass in the definitive histological examination. This latter occurrence would also explain the failure of detection in core biopsy. This recommendation is even more strongly advised for women with a family history of breast cancer.

However, since there is no defined protocol for the treatment of PASH, the different available options need to be extensively discussed by multidisciplinary team and agreed by patients according to familial history, local symptoms, psychological approach and compliance to follow-up.

Among future prospects, we hope that research and study on rare breast lesions [26, 27] such as PASH can continue and expand, as well as on other lesions that often escape clinical and radiological observation but are actually more prevalent than believed. Additionally, conducting multicenter studies to increase the sample size and generate standard guidelines for diagnosing and treating such lesions would be ideal.

## Data Availability

All data generated or analyzed during this study are included in this article.
